# Supplementary material for: A PTEN variant uncouples longevity from impaired fitness in Caenorhabditis elegans with reduced insulin/IGF-1 signaling
Source: Nat Commun. 2021 Sep 24;12:5631. doi: 10.1038/s41467-021-25920-w (PMC8463539; doi:10.1038/s41467-021-25920-w)
Supplement: Supplementary file 1 — Supplementary Information [file 41467_2021_25920_MOESM1_ESM.pdf]

Supporting Information

Supplementary Figures

Supplementary Figure 1

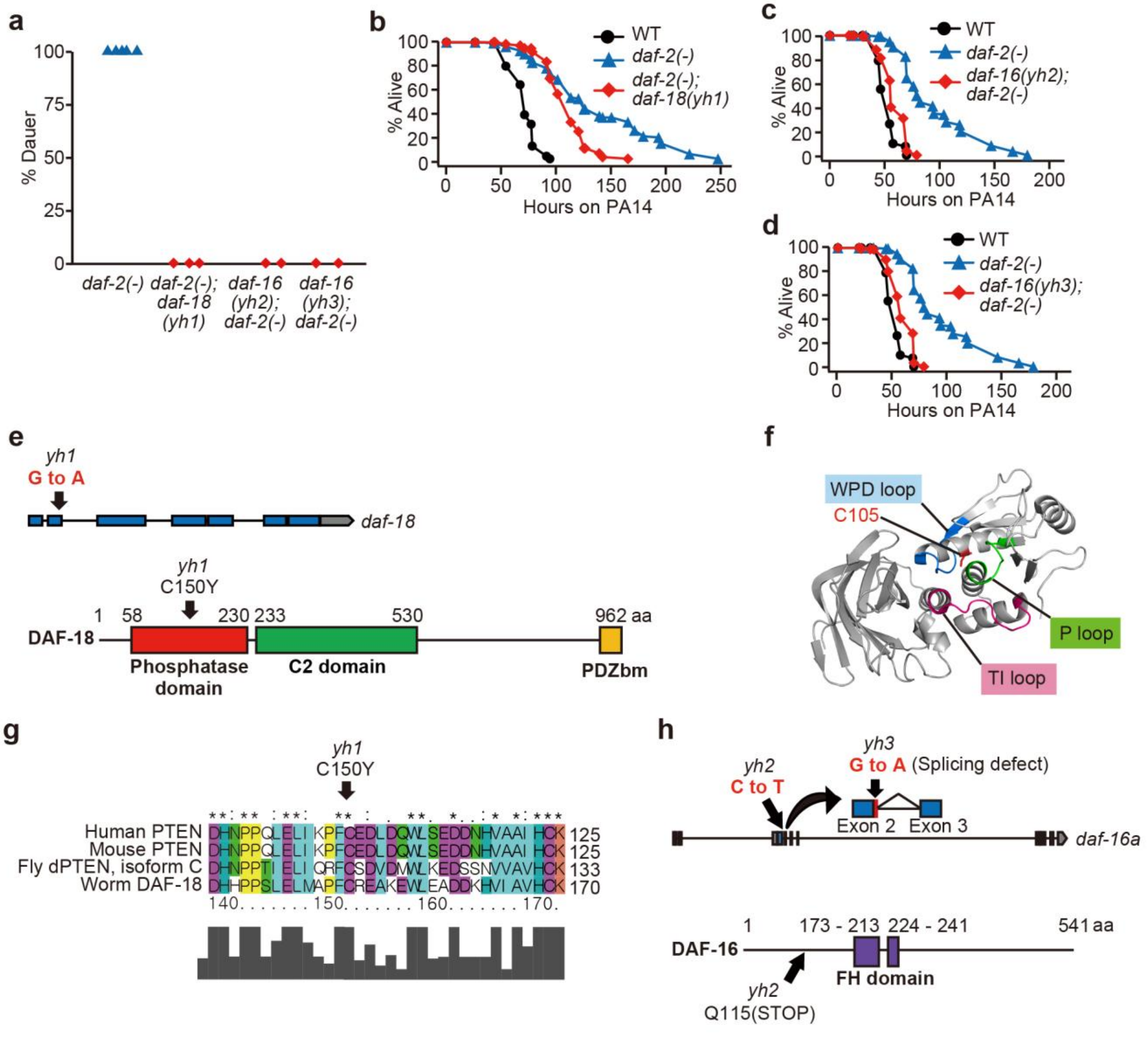

Supplementary Fig. 1: Identification of mutations that suppress dauer phenotypes with small effects on immunity in *daf-2(e1370)* mutants. (**a**) Percentage of 25°C dauer formation of *daf-2(e1370); daf-18(yh1)* [*daf-2(-); daf-18(yh1)*], *daf-16(yh2); daf-2(-)*, and *daf-16(yh3); daf-2(-)* animals, which were identified from our mutagenesis

screen, and *daf-2(-)* animals that were used as a control (n ≥ 267 for each condition, the assays were performed at least twice). (**b**-**d**) Survival curves of *daf-2(-); daf-18(yh1)* (**b**), *daf-16(yh2); daf-2(-)* (**c**), and *daf-16(yh3); daf-2(-)* (**d**) mutants on PA14 compared with *daf-2(-)* mutant and wild-type (WT) worms (n = 180 for each condition). (**e**-**h**) Molecular natures of *yh1*, *yh2*, and *yh3* alleles identified from our mutagenesis screen (See Supplementary Table 1 for details). (**e**) The locus of the *yh1* allele in *daf-18* and the corresponding change in *C. elegans* DAF-18/PTEN protein. *yh1* mutation changes G to A at the 2nd exon of *daf-18*, which causes the cysteine to tyrosine change at the location of 150th amino acid in DAF-18/PTEN. Amino acid (aa) numbers are indicated at the top of the protein domains. Phosphatase domain (58 aa – 230 aa), C2 domain (233 aa – 530 aa), and PDZ-binding motif (PDZbm) are indicated as boxes. (**f**) Three-dimensional structure of human PTEN[1]. C105 resides in a region between two catalytic loops, WPD loop and a highly-conserved core P loop. (**g**) Alignment of amino acid sequences of human PTEN, mouse PTEN, *Drosophila* (fly) dPTEN isoform C, and *C. elegans* (worm) DAF-18. A gray histogram represents the conservation of the sequences. (**h**) The loci of *yh2* and *yh3* alleles in *daf-16* isoform a [*daf-16a*] and the corresponding changes in *C. elegans* DAF-16/FOXO protein. *yh2* causes a nonsense mutation by a C to T transition at the 2nd exon of *daf-16a*, resulting in premature termination at the 115th glutamine. *yh3* causes a G to A transition at the 5' splicing site of intron 2, resulting in a predicted pre-mRNA splicing defect, leading to the acquisition of a premature stop codon. The structural analysis showed that *daf-16(yh2)* and *daf-16(yh3)* mutations may prevent the translation of the forkhead (FH) domain (173 aa - 213 aa and 224 aa - 241 aa) of DAF-16/FOXO due to generation of premature stop codons. See Supplementary Dataset 2

and 3 for additional repeats and statistical analysis for the survival and the dauer assay data shown in this figure. See also Source Data for data points used for the derivation of data.

Supplementary Figure 2

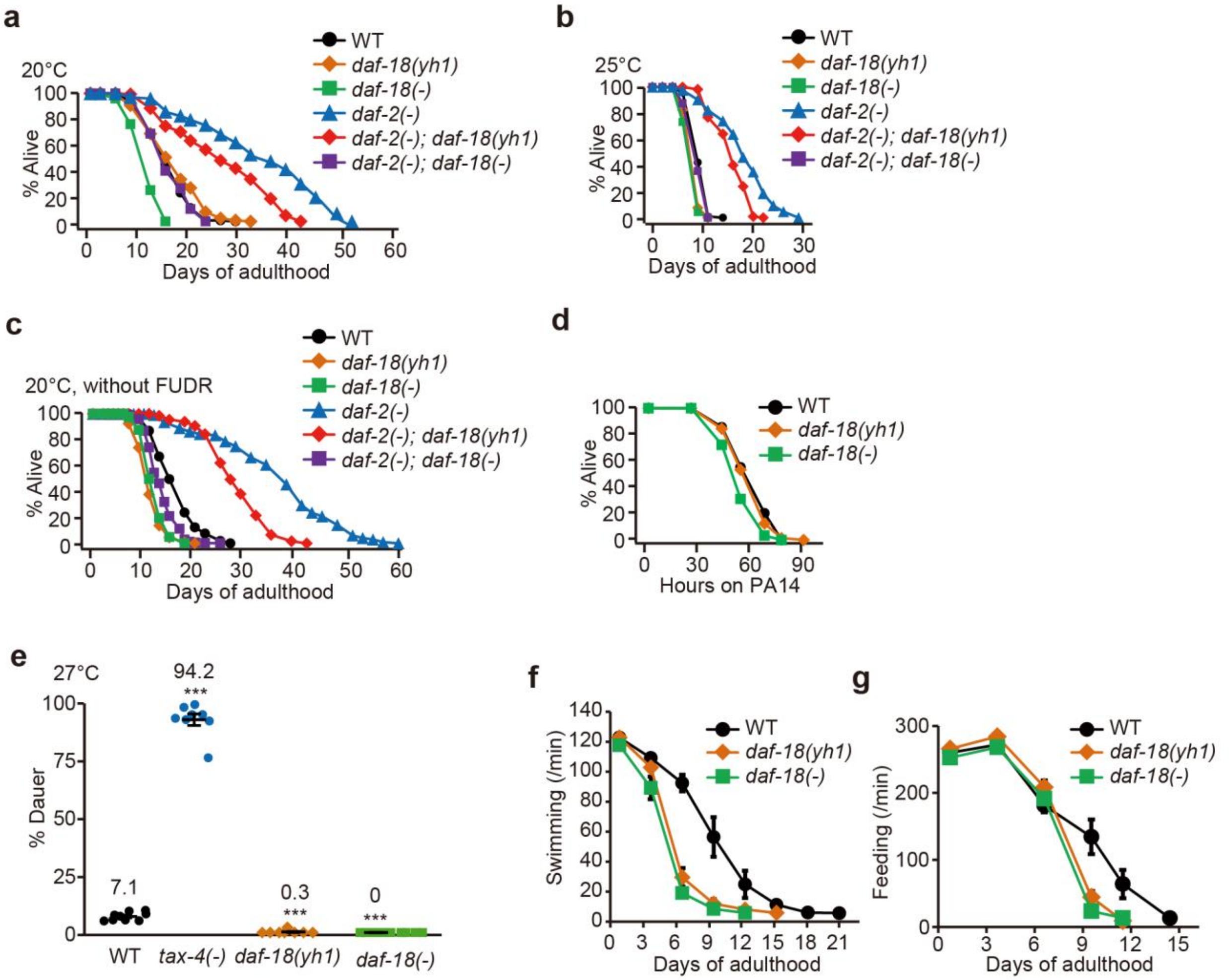

Supplementary Fig. 2: *daf-18(yh1)* decreases pathogen resistance, lifespan, and health span in *daf-2(-)* and wild-type animals. (**a**-**c**) The effects of *daf-18(yh1)* and *daf-18(nr2037)* [*daf-18(-)*] on the lifespan of wild-type (WT) and *daf-2(e1370)* [*daf-2(-)*] animals at 20°C (**a**), at 25°C (**b**), and at 20°C without FUDR treatment (**c**) (n ≥ 225 for each condition). *daf-18(yh1)* substantially decreased the lifespan of WT animals with an extent similar to that caused by *daf-18(-)* at 20°C (four out of six trials). The lifespan curves of WT, *daf-2(-)*, *daf-2(-); daf-18(yh1)*, and *daf-2(-); daf-18(-)* shown in panel **a** are the same experimental sets shown in Fig. 1i, and those in **c** are the same experimental sets shown in Supplementary Fig. 10b and Fig. 6b. (**d**-**g**) The effects of *daf-18(yh1)* and

daf-18(-) on pathogen resistance (n = 180 for each condition) (**d**), dauer formation at 27°C (n ≥ 790 for each condition, from at least eight trials) (**e**), swimming span (n = 10 for each condition, from one trial) (**f**), and feeding span (n = 10 for each condition, from one trial) (**g**). The survival of *daf-18(yh1)* animals under PA14 infection was similar to that of WT worms, whereas *daf-18(-)* worms displayed reduced survival (**d**). Both *daf-18(yh1)* (0.3%) and *daf-18(-)* (0%) mutations substantially suppressed dauer phenotypes at 27°C; WT animals displayed 7.1% of dauers at 27°C, and *tax-4(p678)* [*tax-4(-)*] animals, which display a constitutive dauer phenotype at 27°C[2], was used as a positive control (**e**). In addition, *daf-18(yh1)* and *daf-18(-)* mutations similarly reduced the swimming (motility) and feeding spans (**f**,**g**). Error bars indicate the standard error of mean (s.e.m., ****p* < 0.001, two-tailed Student's *t*-test relative to WT). See Supplementary Dataset 2, 3, and 4 for additional repeats and statistical analysis for the survival, dauer, and health span assay data shown in this figure. See also Source Data for data points used for the derivation of data.

Supplementary Figure 3

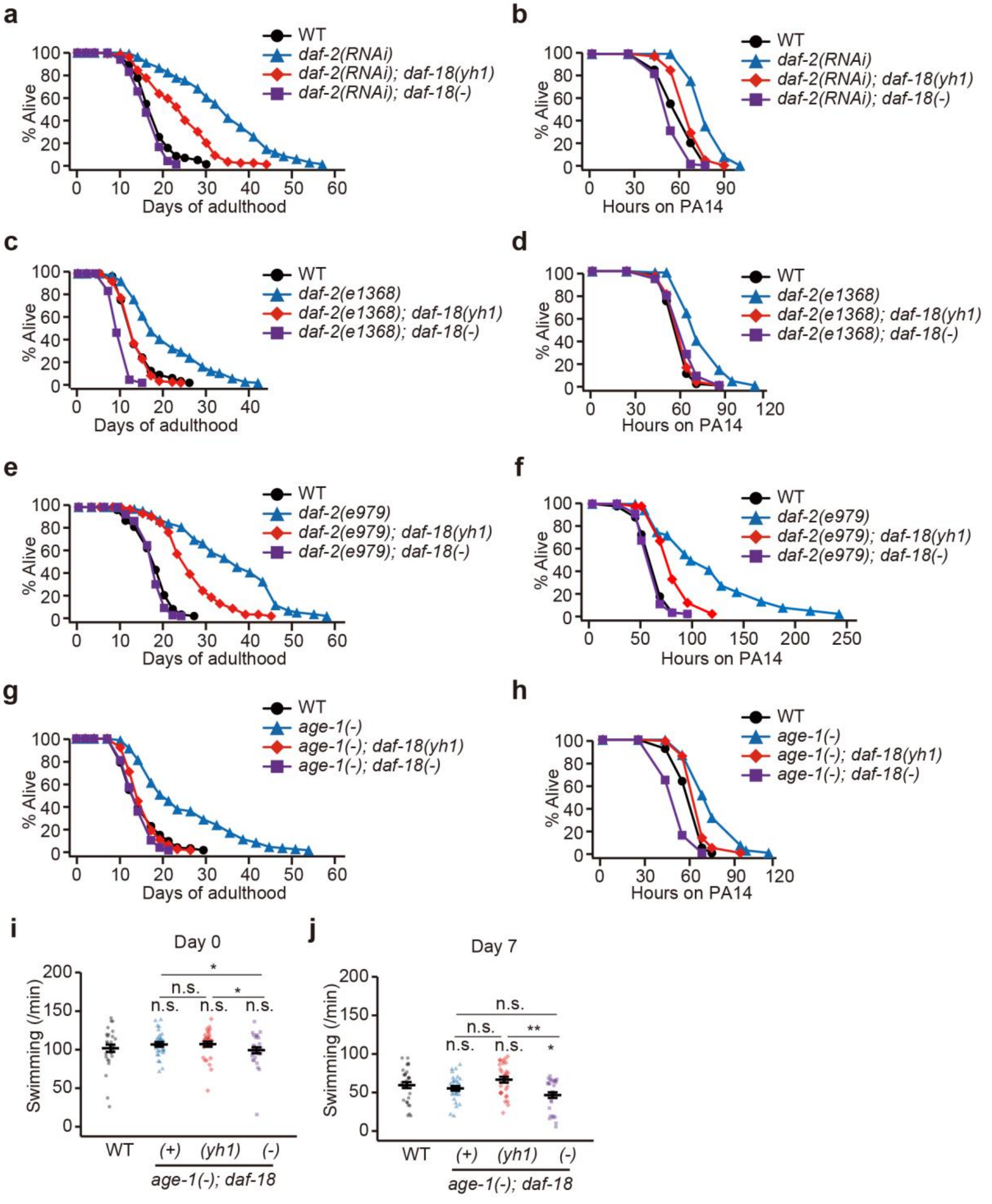

Supplementary Fig. 3: *daf-18(yh1)* retains lifespan and pathogen resistance in worms with reduced IIS in multiple conditions. (**a-h**) The effects of *daf-18(yh1)* and *daf-18(nr2037)* [*daf-18(-)*] on lifespan at 20°C (n ≥ 270 for each condition; n = 156 for

daf-2(e979) animals) and pathogen resistance (n = 180 for each condition; n = 35 for *daf-2(e979)* animals) of *daf-2(RNAi)* (**a**,**b**), *daf-2(e1368)* (**c**,**d**), *daf-2(e979)* (**e**,**f**), and *age-1(hx546)* [*age-1(-)*] (**g**,**h**) animals. (**i**,**j**) Swimming rate (n = 30 for each condition, body bends per minute in liquid measured from three independent trials) of day 0 (**i**) and day 7 adult (**j**) wild-type (WT), *age-1(-); daf-18(+)*, *age-1(-); daf-18(yh1)*, and *age-1(-); daf-18(-)* animals. Error bars indicate the standard error of mean (s.e.m., *$p < 0.05$, **$p < 0.01$, n.s.: not significant, two-tailed Student's *t*-test relative to WT unless otherwise noted). See Supplementary Dataset 2 and 4 for additional repeats and statistical analysis for the survival assay and health span assay data shown in this figure. See also Source Data for data points used for the derivation of data.

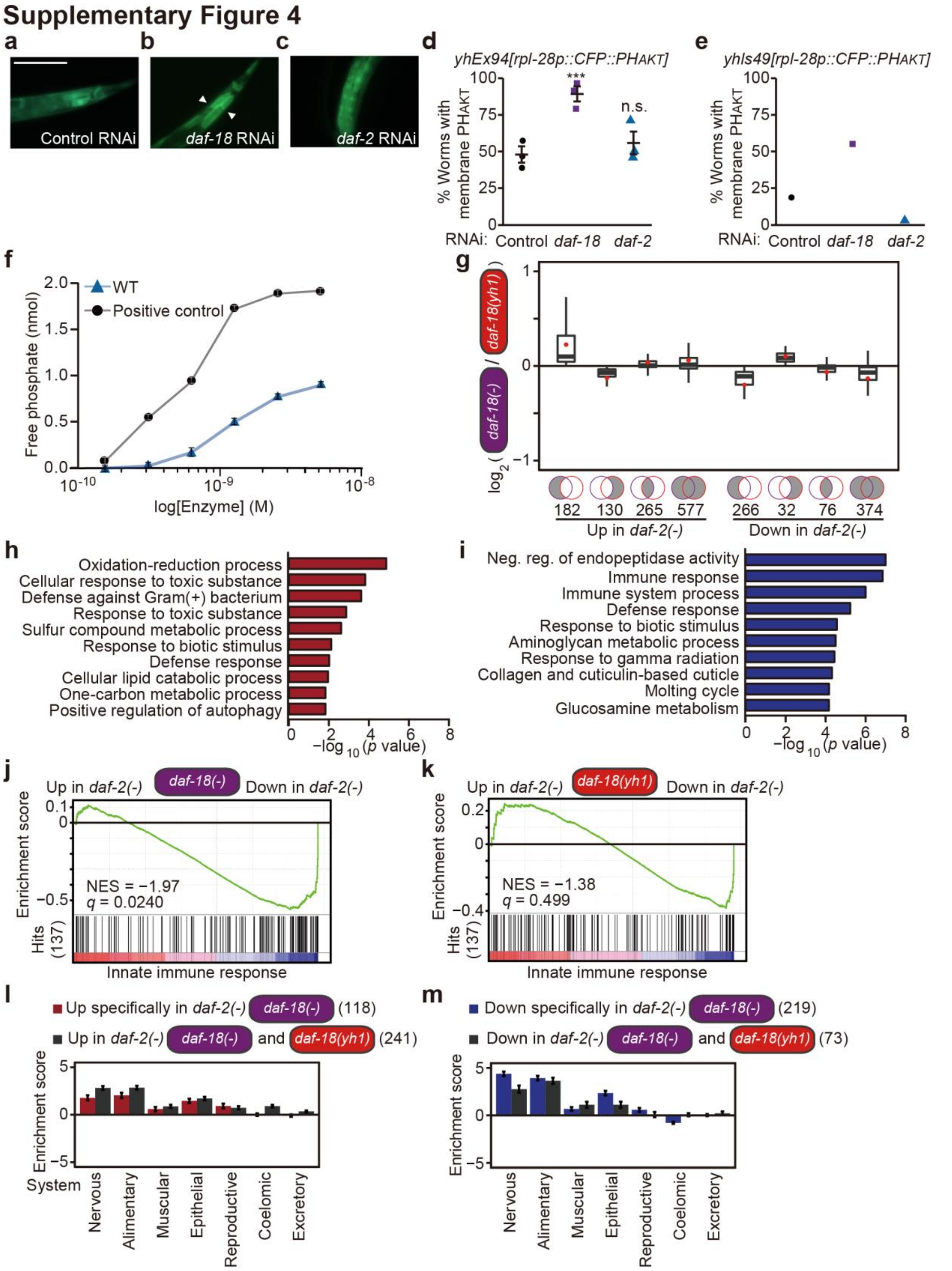

69 **Supplementary Fig. 4: Differential effects of *daf-18(yh1)* and *daf-18(-)* mutations**

on phosphatase activity and various downstream targets in reduced IIS conditions. (**a**-**c**) Subcellular localization of mouse PH_{AKT} for monitoring PIP_3 levels in *C. elegans*. Representative images of *rpl-28p::CFP::*PH_{AKT}-expressing animals treated with control RNAi (**a**), *daf-18* RNAi (**b**), or *daf-2* RNAi (**c**). *rpl-28p* is a ubiquitous *rpl-28* (ribosomal protein large subunit 28) gene promoter. Worms expressing *CFP*::PH_{AKT} as an extrachromosomal array, *yhEx94[rpl-28p::CFP::*PH_{AKT}, *odr-1p::RFP]*. Scale bar: 50 µm. Arrowhead: membrane CFP::PH_{AKT}. (**d**) Quantification of panels **a**-**c** (n > 10 for each condition, from three independent trials). Error bars indicate the standard error of mean (s.e.m., ***$p < 0.001$, n.s.: not significant, two-tailed Student's *t*-test relative to WT). (**e**) Quantification of fluorescent protein levels of integrated CFP::PH_{AKT} worms, *yhIs49[rpl-28p::CFP::*PH_{AKT}*; odr-1p::RFP]* (n > 10 for each condition, from one trial). (**f**) Dose-dependent changes in the protein phosphatase activity of human recombinant PTEN (WT) and protein tyrosine phosphatase 1B (PTP1B, positive control). The indicated amounts of recombinant proteins were tested for the protein phosphatase assay (N = 3). Note that the same dose-dependent protein phosphatase activity curve of WT is shown in Fig. 3h as well. (**g**) Overall magnitudes of gene expression changes caused by *daf-18(yh1)* were smaller than those by *daf-18(nr2037)* [*daf-18(-)*] in *daf-2(e1370)* [*daf-2(-)*] mutants. Venn diagrams with gray area display overlaps between differentially expressed genes. Box plots represent five-number summaries of expression changes of genes that are included in the gray areas in the Venn diagrams. Red dots in the box plots display mean expression changes. (**h**,**i**) Overrepresented gene ontology (GO) terms of the genes that were upregulated (**h**) and downregulated (**i**) in *daf-2(-)* mutants compared specifically with *daf-2(-); daf-18(-)* animals but not with

daf-2(-); daf-18(yh1) worms. *p* values were calculated by using hypergeometric test. (**j**,**k**) Innate immune response genes (curated as a GO term) were significantly induced by *daf-18(-)* (**j**), but not by *daf-18(yh1)* (**k**), in the *daf-2(-)* mutant background. NES: normalized enrichment score. *q* values were obtained by calculating the false discovery rate corresponding to each NES. (**l**) Tissue enrichment analysis of genes upregulated in *daf-2(-)* animals compared specifically with *daf-2(-); daf-18(-)* animals but not with *daf-2(-); daf-18(yh1)* animals, or generally compared with both *daf-2(-); daf-18(-)* and *daf-2(-); daf-18(yh1)* animals. (**m**) Tissue enrichment analysis of genes downregulated in *daf-2(-)* animals compared specifically with *daf-2(-); daf-18(-)* animals but not with *daf-2(-); daf-18(yh1)* animals, or generally compared with both *daf-2(-); daf-18(-)* and *daf-2(-); daf-18(yh1)* animals. See Source Data for data points used for the derivation of data.

Supplementary Figure 5

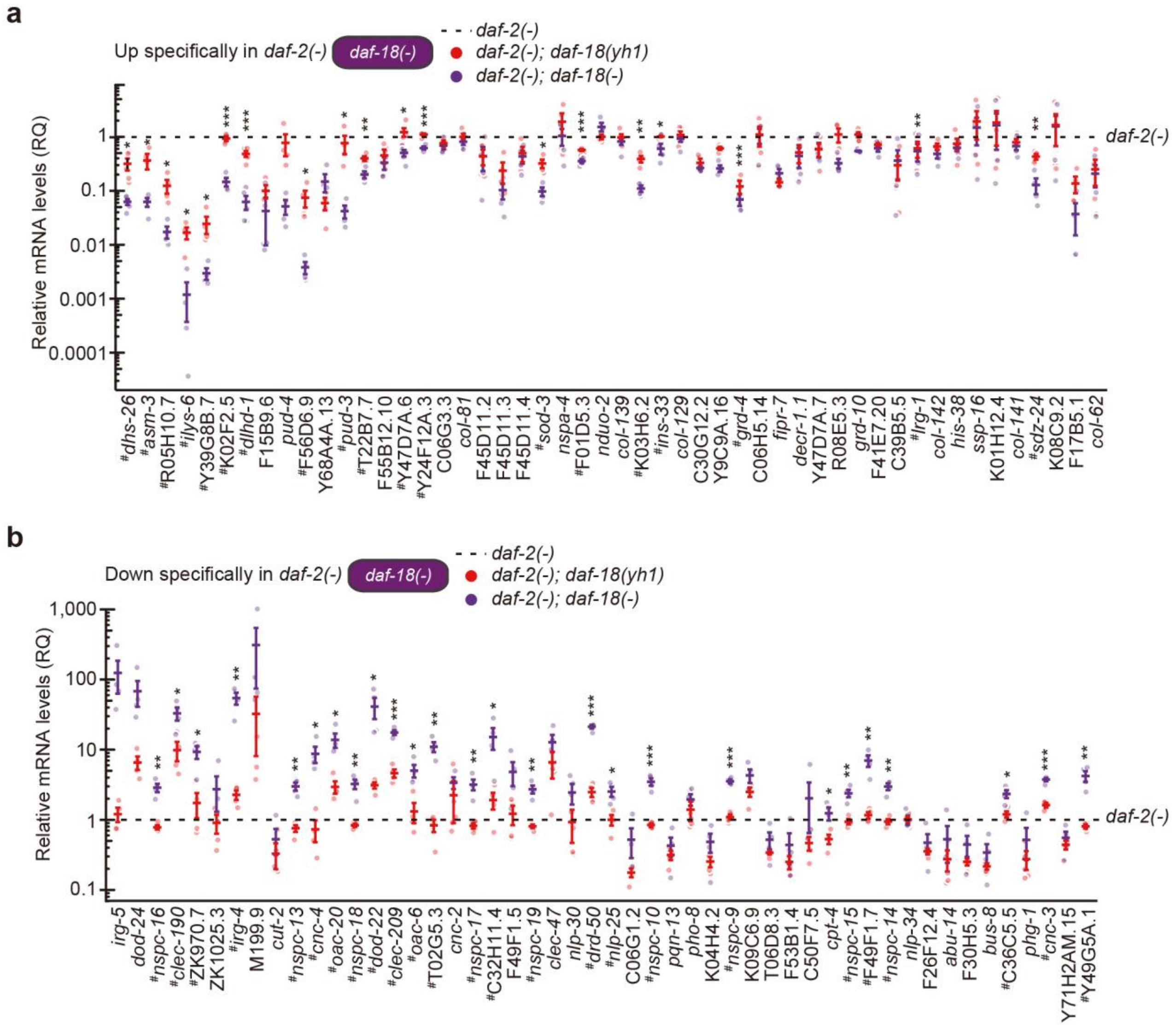

Supplementary Fig. 5: Confirmation of genes whose expression was greatly affected by *daf-18(-)* compared with that by *daf-18(yh1)* in *daf-2(-)* mutants.

Relative mRNA levels of selected genes whose expression was highly affected by *daf-18(nr2037)* [*daf-18(-)*] compared with that by *daf-18(yh1)* in *daf-2(e1370)* [*daf-2(-)*] using quantitative RT-PCR (N ≥ 4). (**a**) Among the top-ranked 50 genes that were upregulated in *daf-2(-)* animals compared with *daf-2(-); daf-18(-)* (fold change > 2, Benjamini and Hochberg (BH)-adjusted *p* value < 0.05), but marginally with *daf-2(-); daf-18(yh1)* worms

(fold change < 2), 19 genes (marked as [#]) displayed the same tendency as RNA-seq data by using qRT-PCR. (**b**) Among the 50 genes that were robustly downregulated in *daf-2(-)* animals compared with *daf-2(-); daf-18(-)* worms (fold change > 2, BH-adjusted p value < 0.05) but slightly with *daf-2(-); daf-18(yh1)* animals (fold change < 2), 25 genes (marked as [#]) displayed the same tendency as RNA-seq data by using qRT-PCR. [#]: *daf-2(-)* vs. *daf-2(-); daf-18(-)* and *daf-2(-); daf-18(yh1)* vs. *daf-2(-); daf-18(-)*, p <0.05. RQ: relative quantity. Black dotted lines indicate the RQ value of *daf-2(-)* animals set as 1. Error bars indicate the standard error of mean (s.e.m., *p < 0.05, **p < 0.01, ***p < 0.001, two-tailed Student's t-test). See Supplementary Dataset 5 for the details of primer sequences. See also Source Data for data points used for the derivation of data.

Supplementary Figure 6

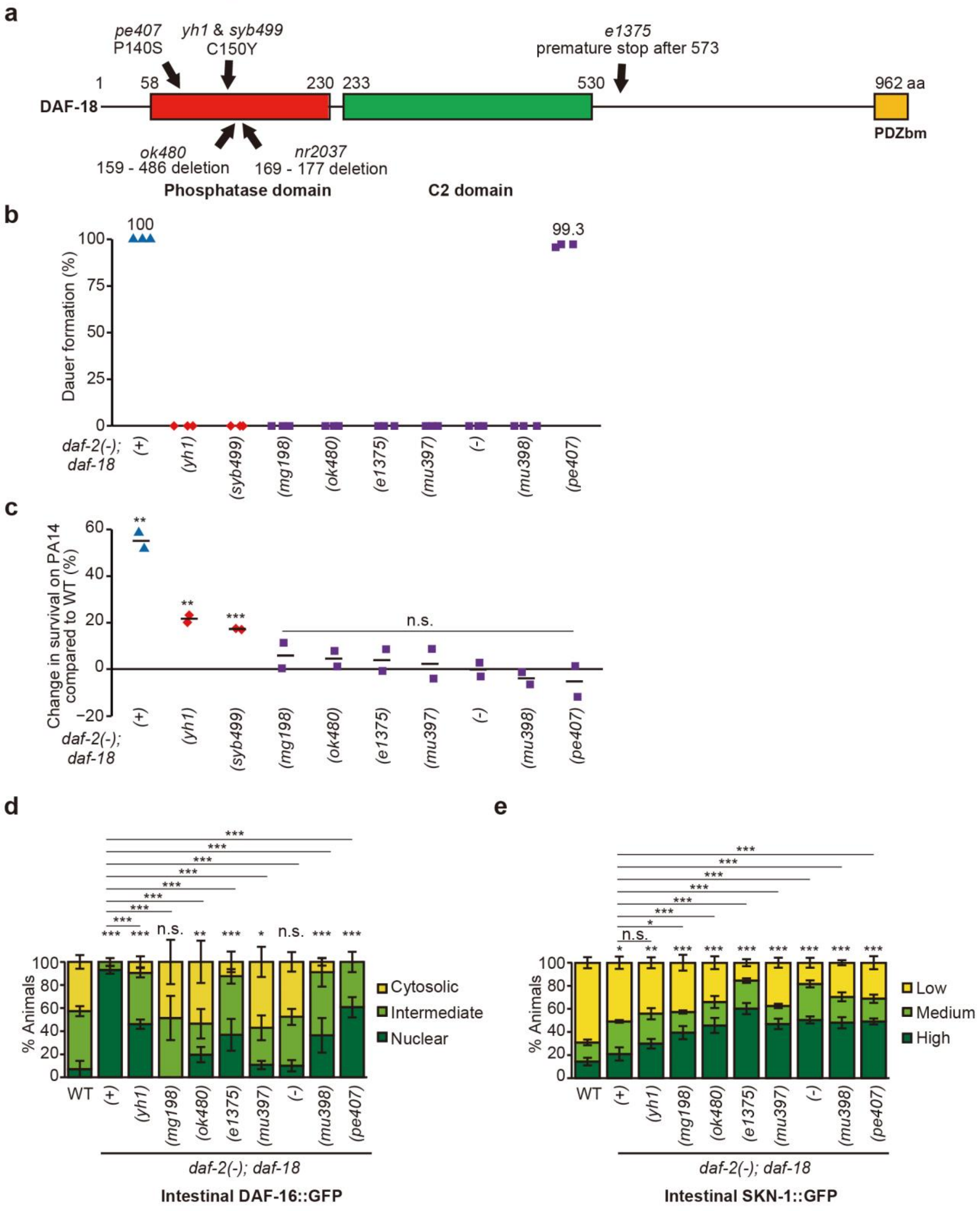

Supplementary Fig. 6: The effects of various *daf-18* mutant alleles on dauer

formation, immunity, and the subcellular localization of DAF-16/FOXO and SKN-1/NRF2 in *daf-2(-)* mutants. (**a**) Locations of *daf-18* hypomorphs that were used for the experiments in the current work[3-10]. *pe407* was isolated from the mutagenesis screen as a suppressor that restores the plasticity of salt chemotaxis in *casy-1(tm718)* mutant backgrounds[10]. *pe407* causes P140S change in the phosphatase domain of DAF-18/PTEN. *yh1* (this study) and *syb499* (CRISPR knock-in version of *yh1*) results in C150Y change, at the phosphatase domain of DAF-18. Amino acid (aa) numbers are indicated at the top of the protein domains. Phosphatase domain, C2 domain, and PDZ-binding motif (PDZbm) are indicated as boxes. *nr2037* (169 aa – 177 aa deletion)[3,4] and *ok480* (159 aa – 486 aa deletion)[7] are deletion alleles of *daf-18*, and *e1375* has a 30 bp insertion that introduces a premature stop codon at 573 aa[5,8]. (**b**,**c**) The effects of various *daf-18* mutant alleles on dauer formation at 25°C (n ≥ 287 for each condition, from three to five independent trials; the assays were performed 96 hrs after eggs were placed) (**b**) and the survival of worms on the pathogen (PA14) using a big-lawn assay (n = 180 for each condition, from two independent trials) (**c**) in the *daf-2(-)* background. Wild-type (WT), *daf-2(e1370)* [*daf-2(-)*], *daf-2(-); daf-18(yh1)*, *daf-2(-); daf-18(syb499)*, *daf-2(-); daf-18(mg198)*, *daf-2(-); daf-18(ok480)*, *daf-2(-); daf-18(e1375)*, *daf-2(-); daf-18(mu397)*, *daf-2(-); daf-18(nr2037)* [*daf-18(-)*], *daf-2(-); daf-18(mu398)*, and *daf-2(-); daf-18(pe407)* animals were examined. Consistent with previous reports[5,8,11], *daf-2(-); daf-18(e1375)* animals developed slowly but eventually became adults at the time dauer formation was assayed. Horizontal lines in panel **c** represent mean values (**$p < 0.01$, ***$p < 0.001$, n.s.: not significant, two-tailed Student's *t*-test relative to WT). (**d**,**e**) The effects of various *daf-18* mutant alleles on the subcellular localization of DAF-16::GFP

and SKN-1::GFP in *daf-2(-)* mutants. (**d**) Quantification of the subcellular localization of DAF-16::GFP in the intestines of indicated strains. Cytosolic: predominant cytosolic localization, intermediate: partial nuclear localization, nuclear: predominant nuclear localization (n ≥ 28 for each condition, from four to seven independent trials). (**e**) Quantification of the subcellular localization of SKN-1::GFP in the intestinal cells of indicated strains. Low: very dim GFP in the nuclei, medium: < 50% of the nuclei with SKN-1::GFP, high: > 50% of the nuclei with SKN-1::GFP (n ≥ 144 for each condition, from four to eight independent trials). The quantification data of DAF-16::GFP subcellular localization in WT, *daf-2(-)*, *daf-2(-); daf-18(yh1)*, and *daf-2(-); daf-18(-)* animals shown in panel **d** are the same experimental sets shown in Fig. 5g, and those in panel **e** are the same experimental sets shown in Fig. 5i. Error bars represent the standard error of mean (s.e.m., *$p < 0.05$, **$p < 0.01$, ***$p < 0.001$, n.s.: not significant, Chi-squared test). See Supplementary Dataset 2 and 3 for additional repeats and statistical analysis for the survival and dauer assay data shown in this figure. See also Source Data for data points used for the derivation of data.

Supplementary Figure 7

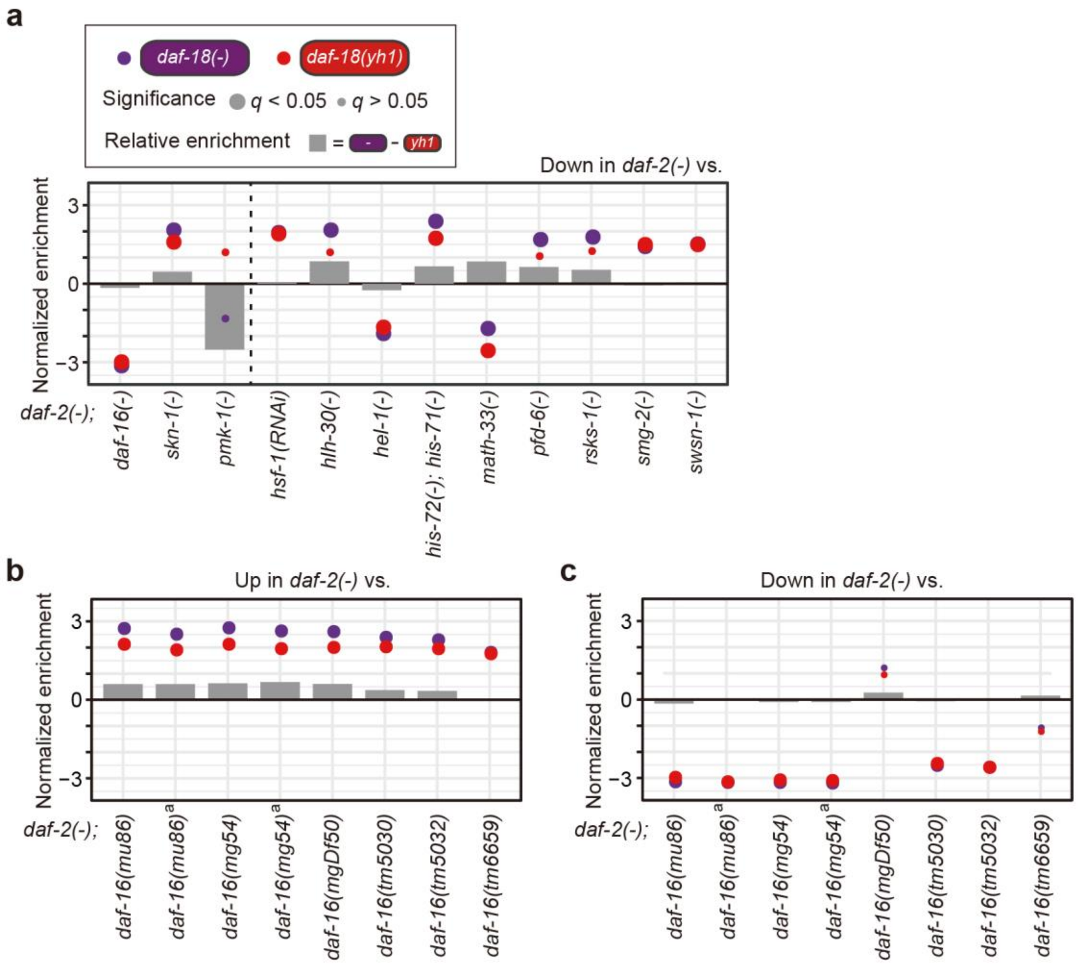

Supplementary Fig. 7: Differential effects of *daf-18(yh1)* and *daf-18(-)* on the expression of various target genes in worms with reduced insulin/IGF-1 signaling. (**a**-**c**) Normalized enrichment of gene expression changes in *daf-2(e1370)* [*daf-2(-)*] mutants compared to *daf-2(-); daf-18(yh1)* or *daf-2(-); daf-18(nr2037)* [*daf-18(-)*] mutants. (**a**) Genes downregulated in *daf-2(-)* animals compared to various lifespan mutants in the *daf-2(-)* background were used for the analysis. Shown are DAF-16/FOXO[12], SKN-1/NRF2[13], PMK-1/p38 MAP kinase[14], HSF-1/heat shock factor 1[15], HLH-30/TFEB[16], HEL-1/DEAD-box RNA helicase[17], histone H3.3[18], MATH-33/deubiquitylating enzyme (at 25°C)[19], PFD-6/prefoldin 6[20], RSKS-1/S6K[21], SMG-

2/UPF1[22], and SWSN-1/BAF155/170[23] target genes. (**b**-**c**) Genes upregulated (**b**) and downregulated (**c**) in *daf-2(-)* animals compared to various alleles of *daf-16* mutants in the *daf-2(-)* background were used for the analysis. Shown are data using *daf-16(mu86)*[12], *daf-16(mu86)*[a], *daf-16(mg54)*[12], *daf-16(mg54)*[a], *daf-16(mgDf50)*[24], *daf-16(tm5030)*[12], *daf-16(tm5032)*[12], and *daf-16(tm6659)*[12]. Relative enrichment indicates the difference of gene expression changes caused by *daf-18(yh1)* or *daf-18(-)*. *q* values were obtained by calculating the false discovery rate corresponding to each normalized enrichment. [a]: GSE111338 in GEO. See Supplementary Table 2 for details.

Supplementary Figure 8

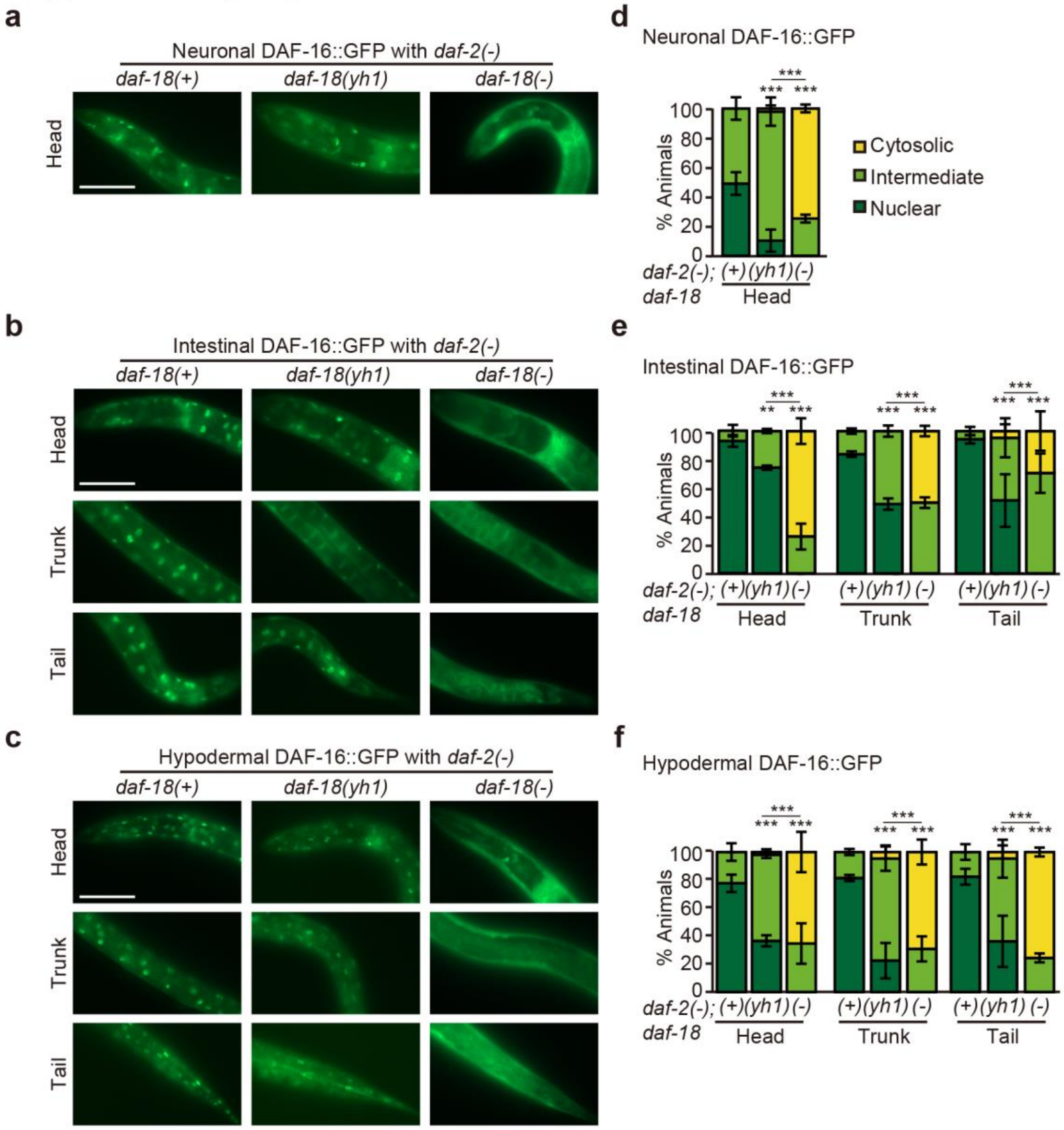

Supplementary Fig. 8: *daf-18(yh1)* partially retains the nuclear localization of DAF-16::GFP in various tissues of *daf-2(-)* mutants. (**a**-**c**) Representative images of the neuronal (**a**), intestinal (**b**), and hypodermal (**c**) DAF-16::GFP localization in head, trunk, and tail regions of *daf-2(e1370)* [*daf-2(-)*], *daf-2(-); daf-18(yh1)*, and *daf-2(-); daf-18(nr2037)* [*daf-18(-)*] animals. Scale bar: 50 μm. (**d**-**f**) Quantification of data shown on panels **a**-**c**. The subcellular localization of DAF-16::GFP was scored as follows. Nuclear:

predominant nuclear localization, intermediate: partial nuclear localization, cytosolic: predominant cytosolic localization ($n \geq 31$ for each condition, from three independent trials). Error bars represent the standard error of mean (s.e.m., **$p < 0.01$, ***$p < 0.001$, Chi-squared test relative to WT unless otherwise noted). See also Source Data for data points used for the derivation of data.

Supplementary Figure 9

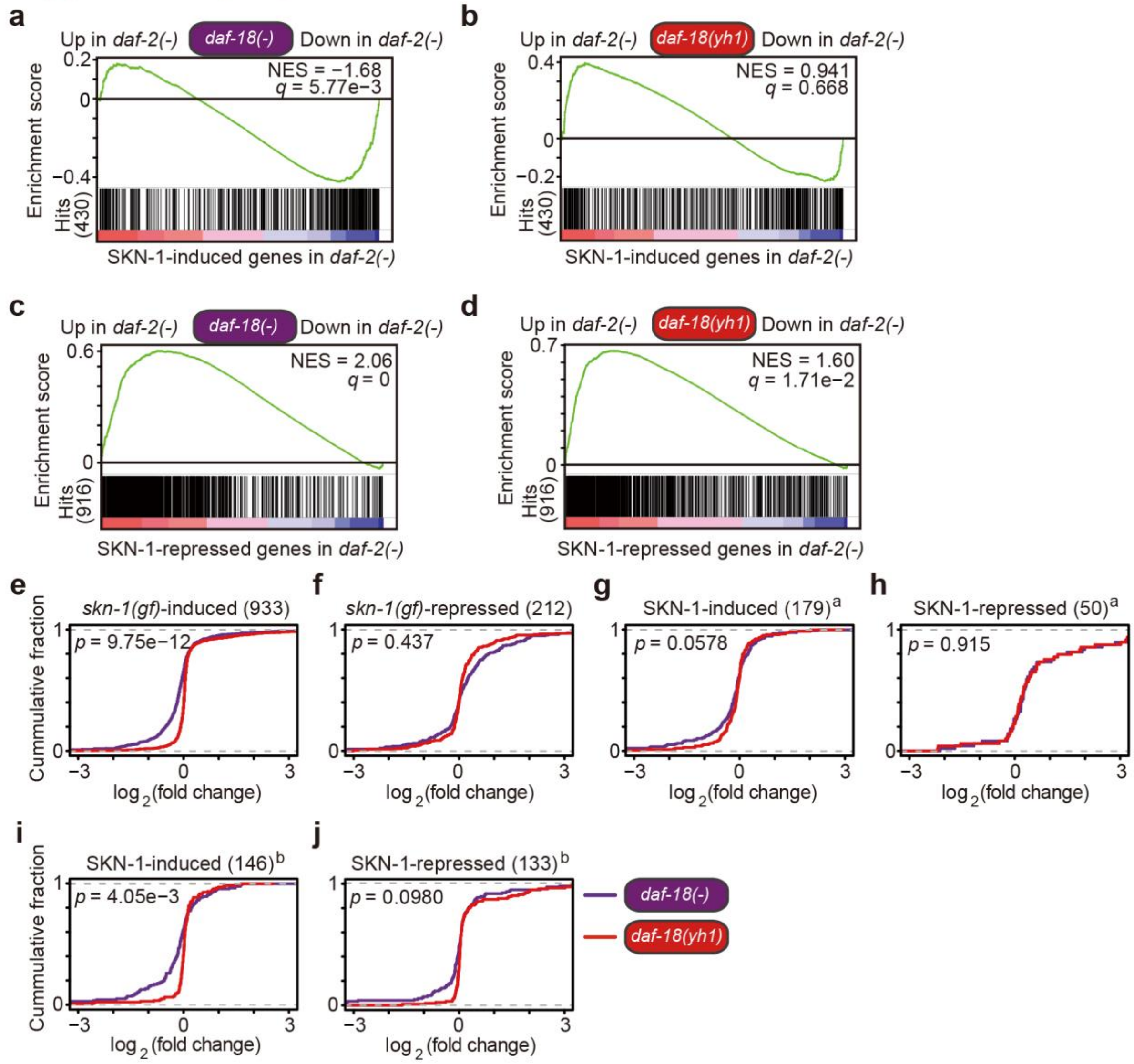

Supplementary Fig. 9: DAF-18/PTEN downregulates SKN-1/NRF2 in animals with reduced insulin/IGF-1 signaling. (**a-d**) The extent of gene expression changes conferred by *daf-18(yh1)* and *daf-18(nr2037)* [*daf-18(-)*] in *daf-2(e1370)* [*daf-2(-)*] animals. Genes whose expression was upregulated (**a**,**b**) and downregulated (**c**,**d**) in *daf-2(e1370)* [*daf-2(-)*] mutants compared to *daf-2(-); skn-1(zu67)* mutants are shown. Data using *daf-2(e1368)* and *daf-2(-)* mutants[13] were pooled. NES: normalized enrichment score. *q* values were obtained by calculating the false discovery rate

corresponding to each NES. (**e**-**j**) Cumulative fraction of genes in an ascending order of the extent of gene expression changes conferred by *daf-18(yh1)* and *daf-18(-)* in wild-type animals. (**e**,**f**) Shown are genes whose expression was upregulated (**e**) and downregulated (**f**) in wild-type worms compared to *skn-1(lax188)* [*skn-1(gf)*] mutants[25]. (**g**-**j**) Genes whose expression was upregulated (**g**,**i**) and downregulated (**h**,**j**) in wild-type worms compared to the worms treated with *skn-1* RNAi are shown [a]: [26]; [b]: [27]. *p* values were calculated by using two-tailed paired permutation test.

Supplementary Figure 10

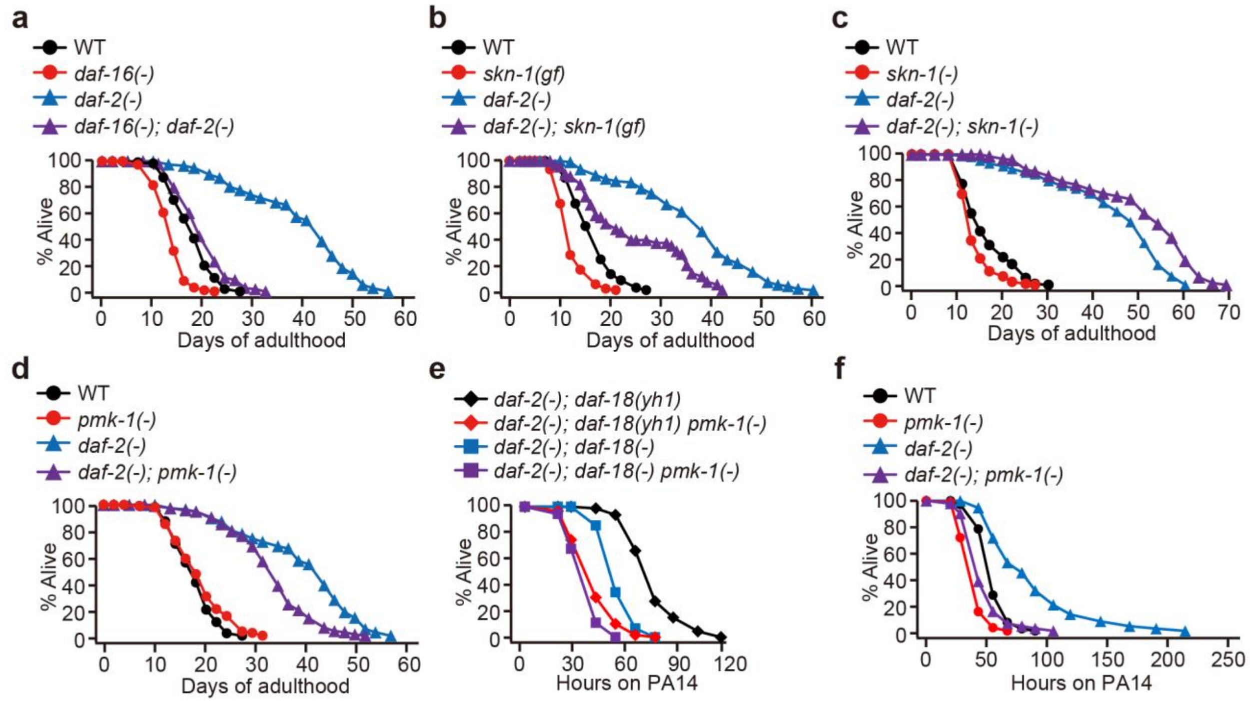

Supplementary Fig. 10: Effects of *daf-16* RNAi, *skn-1(gf)*, *skn-1(-)*, and *pmk-1* RNAi on lifespan and pathogen resistance. (**a**-**c**) Effects of *daf-16* RNAi [*daf-16(-)*] (n = 120 for each condition) (**a**), *skn-1(lax188)* [*skn-1(gf)*] (n ≥ 295 for each condition) (**b**), *skn-1(zj15)* [*skn-1(-)*] (n ≥ 270 for each condition) (**c**), and *pmk-1* RNAi [*pmk-1(-)*] (n = 120 for each condition) (**d**) on the lifespan of wild-type (WT) and *daf-2(e1370)* [*daf-2(-)*] worms. Please note that we used *skn-1(zj15)*, a point mutation that causes mis-splicing and reduces the mRNA levels of *skn-1*, because a strong *skn-1* mutant allele, *skn-1(mg570)*, causes sickness and short lifespan[28]; indeed, we found that *mg570* substantially increased vulval rupture phenotypes in worms: 6% in WT and 22% in *daf-2(-); daf-18(nr2037)* [*daf-18(-)*] backgrounds. (**e**,**f**) The effects of *pmk-1* RNAi (n ≥ 108 for each condition) on the survival of *daf-2(-); daf-18(yh1)* and *daf-2(-); daf-18(-)* (**e**), and WT and *daf-2(-)* worms (**f**) against PA14 infection. Although *daf-2(-); daf-18(-)* animals displayed increased PMK-1 activity compared with *daf-2(-)* or *daf-2(-); daf-18(yh1)*

mutants shown in Fig. 5, reduced DAF-16/FOXO activity in *daf-2(-); daf-18(-)* animals may have decreased the survival of the worms on PA14. These results are consistent with a previous report showing that genetic inhibition of DAF-16/FOXO, which acts downstream of DAF-18/PTEN, increases PMK-1 target gene expression in *daf-2* mutants[14]. In contrast to lifespan results shown in Fig. 6d, we found that PMK-1 was required for the enhanced pathogen resistance of *daf-2(-)* and *daf-2(-); daf-18(yh1)* animals and for the normal survival of WT upon PA14 infection. Our data indicate that reducing the activity of PMK-1 can extend lifespan in *daf-2(-); daf-18(-)* animals, while decreasing immunity in these animals. These data are consistent with a previous report showing that lifespan and immunity can be regulated in opposite directions by one genetic factor[29]. See Supplementary Dataset 2 for statistical analysis and additional repeats for the data shown in this figure. See also Source Data for data points used for the derivation of data.

Supplementary References

1 Rodríguez-Escudero, I. *et al.* A comprehensive functional analysis of PTEN mutations: implications in tumor- and autism-related syndromes. *Hum Mol Genet* **20**, 4132-4142, doi:10.1093/hmg/ddr337 (2011).

2 Ailion, M. & Thomas, J. H. Dauer formation induced by high temperatures in *Caenorhabditis elegans*. *Genetics* **156**, 1047-1067 (2000).

3 Gil, E. B., Malone Link, E., Liu, L. X., Johnson, C. D. & Lees, J. A. Regulation of the insulin-like developmental pathway of *Caenorhabditis elegans* by a homolog of the *PTEN* tumor suppressor gene. *Proc Natl Acad Sci U S A* **96**, 2925-2930, doi:10.1073/pnas.96.6.2925 (1999).

4 Mihaylova, V. T., Borland, C. Z., Manjarrez, L., Stern, M. J. & Sun, H. The PTEN tumor suppressor homolog in *Caenorhabditis elegans* regulates longevity and dauer formation in an insulin receptor-like signaling pathway. *Proc Natl Acad Sci U S A* **96**, 7427-7432, doi:10.1073/pnas.96.13.7427 (1999).

5 Ogg, S. & Ruvkun, G. The *C. elegans* PTEN homolog, DAF-18, acts in the insulin receptor-like metabolic signaling pathway. *Mol Cell* **2**, 887-893, doi:10.1016/s1097-2765(00)80303-2 (1998).

6 Solari, F. *et al.* The human tumour suppressor PTEN regulates longevity and dauer formation in *Caenorhabditis elegans*. *Oncogene* **24**, 20-27, doi:10.1038/sj.onc.1207978 (2005).

7 *C. elegans* Deletion Mutant Consortium, large-scale screening for targeted knockouts in the *Caenorhabditis elegans genome*. *G3 (Bethesda)* **2**, 1415-1425, doi:10.1534/g3.112.003830 (2012).

8 Vowels, J. J. & Thomas, J. H. Genetic analysis of chemosensory control of dauer formation in *Caenorhabditis elegans*. *Genetics* **130**, 105-123 (1992).

9 Lin, K., Hsin, H., Libina, N. & Kenyon, C. Regulation of the *Caenorhabditis elegans* longevity protein DAF-16 by insulin/IGF-1 and germline signaling. *Nat Genet* **28**, 139-145, doi:10.1038/88850 (2001).

10 Ohno, H. *et al.* Role of synaptic phosphatidylinositol 3-kinase in a behavioral learning response in *C. elegans*. *Science* **345**, 313-317, doi:10.1126/science.1250709 (2014).

11 Larsen, P. L., Albert, P. S. & Riddle, D. L. Genes that regulate both development and longevity in *Caenorhabditis elegans*. *Genetics* **139**, 1567-1583 (1995).

12 Chen, A. T. *et al.* Longevity Genes Revealed by Integrative Analysis of Isoform-Specific *daf-16*/*FoxO* Mutants of *Caenorhabditis elegans*. *Genetics* **201**, 613-629, doi:10.1534/genetics.115.177998 (2015).

13 Ewald, C. Y., Landis, J. N., Porter Abate, J., Murphy, C. T. & Blackwell, T. K. Dauer-independent insulin/IGF-1-signalling implicates collagen remodelling in longevity. *Nature* **519**, 97-101, doi:10.1038/nature14021 (2015).

14 Troemel, E. R. *et al.* p38 MAPK regulates expression of immune response genes and contributes to longevity in *C. elegans*. *PLoS Genet* **2**, e183, doi:10.1371/journal.pgen.0020183 (2006).

15 Lee, Y. *et al.* Reduced insulin/IGF1 signaling prevents immune aging via ZIP-

10/bZIP-mediated feedforward loop. *J Cell Biol* **220**, doi:10.1083/jcb.202006174 (2021).

16 Lin, X. X. *et al.* DAF-16/FOXO and HLH-30/TFEB function as combinatorial transcription factors to promote stress resistance and longevity. *Nat Commun* **9**, 4400, doi:10.1038/s41467-018-06624-0 (2018).

17 Seo, M. *et al.* RNA helicase HEL-1 promotes longevity by specifically activating DAF-16/FOXO transcription factor signaling in *Caenorhabditis elegans*. *Proc Natl Acad Sci U S A* **112**, E4246-4255, doi:10.1073/pnas.1505451112 (2015).

18 Piazzesi, A. *et al.* Replication-Independent Histone Variant H3.3 Controls Animal Lifespan through the Regulation of Pro-longevity Transcriptional Programs. *Cell Rep* **17**, 987-996, doi:10.1016/j.celrep.2016.09.074 (2016).

19 Heimbucher, T. *et al.* The Deubiquitylase MATH-33 Controls DAF-16 Stability and Function in Metabolism and Longevity. *Cell Metab* **22**, 151-163, doi:10.1016/j.cmet.2015.06.002 (2015).

20 Son, H. G. *et al.* Prefoldin 6 mediates longevity response from heat shock factor 1 to FOXO in *C. elegans*. *Genes Dev* **32**, 1562-1575, doi:10.1101/gad.317362.118 (2018).

21 Chen, D. *et al.* Germline signaling mediates the synergistically prolonged longevity produced by double mutations in *daf-2* and *rsks-1* in *C. elegans*. *Cell Rep* **5**, 1600-1610, doi:10.1016/j.celrep.2013.11.018 (2013).

22 Son, H. G. *et al.* RNA surveillance via nonsense-mediated mRNA decay is crucial for longevity in *daf-2*/insulin/IGF-1 mutant *C. elegans*. *Nat Commun* **8**, 14749, doi:10.1038/ncomms14749 (2017).

23 Riedel, C. G. *et al.* DAF-16 employs the chromatin remodeller SWI/SNF to promote stress resistance and longevity. *Nat Cell Biol* **15**, 491-501, doi:10.1038/ncb2720 (2013).

24 Kumar, N. *et al.* Genome-wide endogenous DAF-16/FOXO recruitment dynamics during lowered insulin signalling in *C. elegans*. *Oncotarget* **6**, 41418-41433, doi:10.18632/oncotarget.6282 (2015).

25 Nhan, J. D. *et al.* Redirection of SKN-1 abates the negative metabolic outcomes of a perceived pathogen infection. *Proc Natl Acad Sci U S A* **116**, 22322-22330, doi:10.1073/pnas.1909666116 (2019).

26 Oliveira, R. P. *et al.* Condition-adapted stress and longevity gene regulation by *Caenorhabditis elegans* SKN-1/Nrf. *Aging Cell* **8**, 524-541, doi:10.1111/j.1474-9726.2009.00501.x (2009).

27 Steinbaugh, M. J. *et al.* Lipid-mediated regulation of SKN-1/Nrf in response to germ cell absence. *Elife* **4**, doi:10.7554/eLife.07836 (2015).

28 Lehrbach, N. J. & Ruvkun, G. Proteasome dysfunction triggers activation of SKN-1A/Nrf1 by the aspartic protease DDI-1. *Elife* **5**, doi:10.7554/eLife.17721 (2016).

29 Amrit, F. R. G. *et al.* The longevity-promoting factor, TCER-1, widely represses stress resistance and innate immunity. *Nat Commun* **10**, 3042, doi:10.1038/s41467-019-10759-z (2019).

Supplementary Tables

Supplementary Table 1. Molecular natures of mutant alleles identified from our mutagenesis screen.

Allele	*yh1*	*yh2*	*yh3*
Gene	*daf-18*	*daf-16*	*daf-16*
Base change	G to A	C to T	G to A
Function	C150Y	Q115(STOP)	Splice donor
Isoforms affected	All	a/c/d/f/h/i/k/l/m	a/c/d/f/h/i/k/l/m
Domain affected	Phosphatase domain	Forkhead domain	Forkhead domain

Supplementary Table 2. References for multiple target genes in *daf-2* mutants that were used for analysis in Fig. 5 and Supplementary Fig. 7.

Genes	Strains	Figure	Reference
DAF-16-induced/repressed	*daf-2(e1370) vs. daf-16(mu86); daf-2(e1370)*	Fig. 5a and Supplementary Fig. 7a,b	Chen *et al.*, 2015
	daf-2(e1370) vs. *daf-16(mg54); daf-2(e1370)*<br>*daf-2(e1370) vs. daf-16(tm6659); daf-2(e1370)*<br>*daf-2(e1370) vs. daf-16(tm5032); daf-2(e1370)*<br>*daf-2(e1370) vs. daf-16(tm5030); daf-2(e1370)*	Supplementary Fig. 7b,c	
	daf-2(e1370) vs. *daf-16(mu86); daf-2(e1370)*<br>*daf-2(e1370) vs. daf-16(mg54); daf-2(e1370)*	Supplementary Fig. 7b,c	GSE111338 in GEO
	daf-2(e1370) vs. *daf-16(mgDf50); daf-2(e1370)*	Supplementary Fig. 7b,c	Kumar *et al.*, 2015
SKN-1-induced/repressed	*daf-2(e1370)* vs. *daf-2(e1370); skn-1(zu67)*<br>*daf-2(e1368)* vs. *daf-2(e1368); skn-1(zu67)*	Fig. 5a and Supplementary Fig. 7a	Ewald *et al.*, 2015
PMK-1-induced/repressed	*daf-2(e1368)* vs. *daf-2(e1368)*; *pmk-1(km25)*	Fig. 5a and Supplementary Fig. 7a	Troemel *et al.*, 2006

HSF-1-induced/repressed	*daf-2(e1370)* vs. *hsf-1(RNAi); daf-2(e1370)*	Fig. 5a and Supplementary Fig. 7a	Lee *et al.*, 2021
HLH-30-induced/repressed	*daf-2(e1370)* vs. *daf-2(e1370); hlh-30(tm1978)*	Fig. 5a and Supplementary Fig. 7a	Lin *et al.*, 2018
HEL-1-induced/repressed	*daf-2(e1370)* vs. *hel-1(gk148684); daf-2(e1370)*	Fig. 5a and Supplementary Fig. 7a	Seo *et al.*, 2015
Histone H3.3-induced/repressed	*daf-2(e1370)* vs. *daf-2(e1370); his-72(tm2066); his-71(ok2289)*	Fig. 5a and Supplementary Fig. 7a	Piazzesi *et al.*, 2016
MATH-33-induced/repressed	*daf-2(e1370)* vs. *daf-2(e1370); math-33(tm3561)* at 25°C (restrictive temperature)	Fig. 5a and Supplementary Fig. 7a	Heimbucher *et al.*, 2015
PFD-6-induced/repressed	*daf-2(e1370)* vs. *pfd-6(gk493446); daf-2(e1370)*	Fig. 5a and Supplementary Fig. 7a	Son *et al.*, 2018
RSKS-1-induced/repressed	*daf-2(e1370)* vs. *daf-2(e1370); rsks-1(ok1255)*	Fig. 5a and Supplementary Fig. 7a	Chen *et al.*, 2013
SMG-2-induced/repressed	*daf-2(e1370)* vs. *smg-2(qd101); daf-2(e1370)*	Fig. 5a and Supplementary Fig. 7a	Son *et al.*, 2017
SWSN-1-induced/repressed	*daf-2(e1370)* vs. *daf-2(e1370); swsn-1(os22)*	Fig. 5a and Supplementary Fig. 7a	Riedel *et al.*, 2013

320
