# Supplementary material for: A PTEN variant uncouples longevity from impaired fitness in Caenorhabditis elegans with reduced insulin/IGF-1 signaling
Source: Nat Commun. 2021 Sep 24;12:5631. doi: 10.1038/s41467-021-25920-w (PMC8463539; doi:10.1038/s41467-021-25920-w)
Supplement: Supplementary file 3 — Description of Additional Supplementary Files [file 41467_2021_25920_MOESM3_ESM.docx]

**Description of Additional Supplementary Files**

**Title: Supplementary Dataset 1**

Description: Genes whose expression was upregulated and downregulated in *daf-2(e1370)* mutants compared with *daf-2(e1370); daf-18(nr2037)* and/or with in *daf-2(e1370); daf-18(yh1)* worms (XLSX 219 kb).

**Title: Supplementary Dataset 2**

Description: Statistical analysis of survival assay data and additional repeats (XLSX 42 kb).

**Title: Supplementary Dataset 3**

Description: Statistical analysis of dauer assay data and additional repeats (XLSX 16 kb).

**Title: Supplementary Dataset 4**

Description: Statistical analysis of swimming and feeding rate assay data and additional repeats (XLSX 22 kb).

**Title: Supplementary Dataset 5**

Description: List of qRT-PCR primers used in this study (XLSX 20 kb).
