# Supplementary material for: A PTEN variant uncouples longevity from impaired fitness in Caenorhabditis elegans with reduced insulin/IGF-1 signaling
Source: Nat Commun. 2021 Sep 24;12:5631. doi: 10.1038/s41467-021-25920-w (PMC8463539; doi:10.1038/s41467-021-25920-w)
Supplement: Supplementary file 9 — Reporting Summary [file 41467_2021_25920_MOESM9_ESM.pdf]

nature research

Corresponding author(s): Seung-Jae V. Lee

Last updated by author(s): Jul 16, 2021

Reporting Summary

Nature Research wishes to improve the reproducibility of the work that we publish. This form provides structure for consistency and transparency in reporting. For further information on Nature Research policies, see our Editorial Policies and the Editorial Policy Checklist.

Statistics

For all statistical analyses, confirm that the following items are present in the figure legend, table legend, main text, or Methods section.

n/a	Confirmed	
☐	☒	The exact sample size (n) for each experimental group/condition, given as a discrete number and unit of measurement
☐	☒	A statement on whether measurements were taken from distinct samples or whether the same sample was measured repeatedly
☐	☒	The statistical test(s) used AND whether they are one- or two-sided<br>*Only common tests should be described solely by name; describe more complex techniques in the Methods section.*
☐	☒	A description of all covariates tested
☐	☒	A description of any assumptions or corrections, such as tests of normality and adjustment for multiple comparisons
☐	☒	A full description of the statistical parameters including central tendency (e.g. means) or other basic estimates (e.g. regression coefficient) AND variation (e.g. standard deviation) or associated estimates of uncertainty (e.g. confidence intervals)
☐	☒	For null hypothesis testing, the test statistic (e.g. F, t, r) with confidence intervals, effect sizes, degrees of freedom and P value noted<br>*Give P values as exact values whenever suitable.*
☐	☒	For Bayesian analysis, information on the choice of priors and Markov chain Monte Carlo settings
☐	☒	For hierarchical and complex designs, identification of the appropriate level for tests and full reporting of outcomes
☐	☒	Estimates of effect sizes (e.g. Cohen's d, Pearson's r), indicating how they were calculated

Our web collection on statistics for biologists contains articles on many of the points above.

Software and code

Policy information about availability of computer code

Data collection	Relevant data were manually searched in the Gene Expression Omnibus (https://www.ncbi.nlm.nih.gov/geo) and PUMAdb (https://puma.princeton.edu/), if necessary.
Data analysis	Survival assays<br>OASIS (https://sbi.postech.ac.kr/oasis/) and OASIS2 (https://sbi.postech.ac.kr/oasis2/) were used for a log-rank (Manel-Cox method) test.<br><br>Analysis of RNA-sequencing data<br>STAR (v.2.7.0e), RSEM (v.1.3.1), RUVSeq (v1.16.1), GSEA (v.3.0), DESeq2 (v.1.22.2), GOstats (v.2.48.0), Revigo, Worm tissue (https://worm.princeton.edu/), and R (v.3.6.1) were used to analyze data in this study.<br>Sequencing pairs were aligned to the C. elegans genome WBcel235 (ce11) and Ensembl transcriptome (release 95) by using STAR (v.2.7.0e). Aligned pairs on genes were quantified by using RSEM (v.1.3.1). Alignment and quantification of RNA-seq data in this study was conducted based on the parameters described in the guidelines of ENCODE long RNA-Seq processing pipeline (https://www.encodeproject.org/pipelines/ENCPL002LPE/). The batch effects of samples were removed by upper-quartile normalization followed by RUVSeq (v1.16.1) with internal control genes. Global expression changes of gene sets were represented as normalized enrichment scores (NES) by using gene set enrichment analysis (GSEA) (v.3.0) or calculating cumulative fractions with read counts of all expressed genes. Differentially expressed genes were identified by using DESeq2 (v.1.22.2). Gene ontology terms enriched in genes of interest were identified by using GOstats (v.2.48.0), and summarized by using Revigo (http://revigo.irb.hr/about.jsp). These genes were compared to the genes expressed in different tissues based on Worm tissue (https://worm.princeton.edu/). R (v.3.6.1, http://www.r-project.org) was used for all the data plotting in this study.

For manuscripts utilizing custom algorithms or software that are central to the research but not yet described in published literature, software must be made available to editors and reviewers. We strongly encourage code deposition in a community repository (e.g. GitHub). See the Nature Research guidelines for submitting code & software for further information.

Data

Policy information about availability of data

All manuscripts must include a data availability statement. This statement should provide the following information, where applicable:

- Accession codes, unique identifiers, or web links for publicly available datasets
- A list of figures that have associated raw data
- A description of any restrictions on data availability

RNA-seq datasets were deposited at Gene Expression Omnibus under accession number GSE154338.

Field-specific reporting

Please select the one below that is the best fit for your research. If you are not sure, read the appropriate sections before making your selection.

☒ Life sciences ☐ Behavioural & social sciences ☐ Ecological, evolutionary & environmental sciences

For a reference copy of the document with all sections, see nature.com/documents/nr-reporting-summary-flat.pdf

Life sciences study design

All studies must disclose on these points even when the disclosure is negative.

Sample size	All the experiments were performed with at least two independent trials. Standard deviations were calculated from the biological replicates.
Data exclusions	No data were excluded from the study.
Replication	The findings in this study were highly reproducible and all data were from at least two independent biological replicates.
Randomization	The samples were randomly allocated into experimental groups.
Blinding	All subcellular localization assays were performed double-blindly by at least two independent researchers.<br>The other experiments including survival assays in this study were not performed blindly due to clear phenotypic differences between the strains.

Reporting for specific materials, systems and methods

We require information from authors about some types of materials, experimental systems and methods used in many studies. Here, indicate whether each material, system or method listed is relevant to your study. If you are not sure if a list item applies to your research, read the appropriate section before selecting a response.

Materials & experimental systems

n/a	Involved in the study	
☐	☒	Antibodies
☐	☒	Eukaryotic cell lines
☒	☐	Palaeontology and archaeology
☐	☒	Animals and other organisms
☒	☐	Human research participants
☒	☐	Clinical data
☒	☐	Dual use research of concern

Methods

n/a	Involved in the study	
☒	☐	ChIP-seq
☒	☐	Flow cytometry
☒	☐	MRI-based neuroimaging

Antibodies

Antibodies used	Primary antibodies: His antibody (#2365, Cell signaling technology, Danvers, MA, USA),<br>phospho-PMK-1 antibody (#9211, Cell Signaling Technology, Danvers, MA, USA),<br>α-Tubulin (sc-32293, Santa Cruz Biotechnology, Dallas, TX, USA)<br>Secondary antibodies: anti-rabbit (#SA8002, ABfrontier, Seoul, South Korea), anti-mouse (#SA8001, ABfrontier, Seoul, South Korea)
Validation	Anti-His (#2365, Cell Signaling Technology, Danvers, MA, USA) has been extensively validated by using Western blotting and immunoprecipitation. The supplier website (https://www.cellsignal.com/products/primary-antibodies/his-tag-antibody/2365) provides 81 references that have used this antibody for Western blotting, immunoprecipitation, immunocytochemistry and immunofluorescence. We have used this antibody to show that expression of his-tag PTEN protein in sf9 cells by Western blotting.<br>Anti-phospho-p38 MAPK (Thr180/Tyr182) (#9211, Cell Signaling Technology, Danvers, MA, USA) has been validated by using Western blotting. The supplier website (https://www.cellsignal.com/products/primary-antibodies/phospho-p38-mapk-thr180-tyr182-antibody/9211) provides 1972 references for this antibody. In addition, we used α-Tubulin (sc-32293, Santa Cruz Biotechnology,

Dallas, TX, USA) antibody, and the supplier website (https://www.citeab.com/antibodies/835078-sc-32293-anti-tubulin-antibody-dm1a) provides 9 references for validating this antibody. We have used these antibodies and secondary antibodies against rabbit (ABfrontier #SA8002) and mouse (ABfrontier #SA8001) antibodies to measure the levels of phospho-PMK-1 in worms by using Western blotting. The supplier websites [(http://www.younginfrontier.com/laboratory/ab_product/item.php?catalog_no=29633&com_code=&ca_id=&sc_keyword=&sc_brand=&page=21&banner_idx=4) for anti-rabbit, and (http://www.younginfrontier.com/laboratory/ab_product/item.php?catalog_no=18307) for anti-mouse antibodies] provides information of each antibody.

Eukaryotic cell lines

Policy information about cell lines

Cell line source(s)	sf9 cells are gift from Dr. Ji-Joon Song laboratory at KAIST, South Korea.
Authentication	The cells were not authenticated.
Mycoplasma contamination	The cell lines were not tested for mycoplasma.
Commonly misidentified lines (See ICLAC register)	No misidentified lines were used in this study.

Animals and other organisms

Policy information about studies involving animals; ARRIVE guidelines recommended for reporting animal research

Laboratory animals	Larval and adult stages of Caenorhabditis elegans hermaphrodites were used in this study. These include wild-type N2 (Bristol), CF1041, CF1085, CF1042, IJ713, IJ1592, IJ1417, IJ1418, IJ1591, IJ773, IJ681, IJ1854, IJ1646, IJ1554, CF2380, IJ385, IJ1665, IJ2072, IJ1926, IJ1349, IJ1353, TJ1052, IJ1993, IJ1089, IJ604, IJ531, IJ617, IJ264, IJ1855, IJ1856, CF1184, CF1185, IJ484, IJ798, IJ1357, IJ1869, IJ1868, IJ1058, IJ1456, IJ1831, IJ1573, IJ1108, IJ1112, IJ1139, IJ1154, IJ1157, IJ1160, IJ1553, IJ1566, IJ1816, IJ1570, IJ1013, IJ1046, IJ1070, IJ1078, IJ1084, IJ1086, IJ981, IJ1982, IJ1983, IJ1984, IJ1985, IJ1625, IJ2037, IJ2042, IJ921, and IJ1671 (listed based on approved CGC-nomenclature).
Wild animals	No wild animals were used in this study.
Field-collected samples	No field-collected samples were used in this study.
Ethics oversight	N/A

Note that full information on the approval of the study protocol must also be provided in the manuscript.
